# Facile Synthesis of a Novel Hierarchical ZSM‐5 Zeolite: A Stable Acid Catalyst for Dehydrating Glycerol to Acrolein

**DOI:** 10.1002/cctc.201700663

**Published:** 2017-10-16

**Authors:** Rolf Beerthuis, Liang Huang, N. Raveendran Shiju, Gadi Rothenberg, Wei Shen, Hualong Xu

**Affiliations:** ^1^ Van't Hoff Institute for Molecular Sciences University of Amsterdam P.O. Box 94157 1090GD Amsterdam The Netherlands; ^2^ Department of Chemistry Shanghai Key Laboratory of Molecular Catalysis and Innovative Materials Laboratory of Advanced Materials and Collaborative Innovation Center of Chemistry for Energy Materials Fudan University Shanghai 200433 P.R. China

**Keywords:** coking resistance, glycerol dehydration, hierarchical porosity, mesoporosity, zeolites

## Abstract

Catalytic biomass conversion is often hindered by coking. Carbon compounds cover active surface and plug pores, causing catalyst deactivation. Material design at the nanoscale allows tailoring of the catalytic activity and stability. Here, we report a simple synthesis of nanosized ZSM‐5 materials by using a silicalite‐1 seeding suspension. ZSM‐5 crystals were grown from a deionized silica source in the presence of ammonia. By using silicalite‐1 seeds, crystalline ZSM‐5 is synthesized without any structure‐directing agent. This method allows parallel preparation of a range of ZSM‐5 samples, eliminating time‐consuming ion‐exchange steps. Mesoporosity is introduced by formation of intercrystallite voids, owing to nanocrystal agglomeration. The effects of crystal sizes and morphologies are then evaluated in the catalytic dehydration of glycerol to acrolein, with results compared against commercial ZSM‐5. The most active nanosized ZSM‐5 catalysts were five times more stable compared with commercial ZSM‐5, giving quantitative conversion and twice the acrolein yield compared with the commercial catalyst. The influence of the catalyst structure on the chemical diffusion and the resistance to coking are discussed.

## Introduction

Converting biomass to useful chemicals often requires a fresh look at the catalytic process steps, because unlike crude oil, which is under‐functionalized, biomass is over‐functionalized. One of the thorniest problems in this respect is catalyst deactivation through coking. Coke typically plugs the pores and channels of the catalyst, reducing the number of approachable active sites. It can also hamper product selectivity, owing to the difference between the sites “in the pores” and those on the external catalyst surface.^1^, ^2^ Coking is a universal problem, which applies to both supported catalysts and bulk‐phase ones. It is especially acute for zeolite catalysts because of their narrow interconnected channels and pores. The industrial importance of this problem is reflected by the zeolite catalysts market size, which topped one billion US$ in 2014.^3^

**Figure 1:**
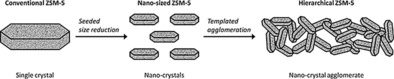
Graphic showing the synthesis concept of hierarchical porous zeolites by using agglomeration through crystal size reduction and further increased porosity owing to templating.

One way to solve this problem is by designing hierarchical porous zeolite structures. Although zeolite synthesis is a mature field, hierarchical zeolites are relative newcomers, attracting much attention.^4^, ^5^ Studies typically distinguish between *inter*crystalline and *intra*crystalline hierarchy.^6^, ^7^ The former consists of mesoporous voids, generated by fragmentation and agglomeration of microporous crystals. The latter involves introducing mesopores within the microporous crystals themselves. Typically, hierarchical zeolites are made either by templating methods or by de‐metalation and controlled crystallization.^4^ Pérez‐Ramirez et al. suggested the distinction between bottom‐up (templating) and top‐down (desilication) approaches to introduce mesoporosity in (alumina)silicates.^4^ Here, we developed a different approach that uses extended agglomeration by decreasing crystal size. This introduces “intercrystalline pores”, increasing the catalyst's porosity and resistance to coking in the dehydration of glycerol to acrolein (see Figure 1).

ZSM‐5 is an especially important example. This porous and highly acidic aluminosilicate is widely used as a catalyst in the petrochemical industry.^2^ Indeed, its development by Mobil Oil is considered a milestone in the zeolite field.^8^ Recently, we showed that using nanosized Au/ZSM‐5 prevents coking in the methanol‐to‐propylene reaction.^9^ Elsewhere, Koekkoek et al. showed that using hierarchical Fe/ZSM‐5 improved the catalytic activity in the hydroxylation of benzene to phenol.^10^ Similarly, Yu et al. demonstrated enhanced selectivity and coke reduction if using hierarchical ZSM‐11 in catalytic pyrolysis of heavy oils.^11^ In the latter example, the nanosized ZSM‐11 particles were prepared by using tetrabutylammonium bromide. Recently, Wang et al. highlighted the importance of incorporating mesopores into microporous ZSM‐5 in the cracking of *n*‐hexane.^12^ The interconnected pore systems shortened the average diffusion path length, which significantly increased catalyst lifetime. Similarly, Bai and co‐workers found high selectivity towards dimethyl ether in methanol conversion at low temperatures, which was ascribed to enhanced mass transport in hierarchical ZSM‐5 pores.^13^ Elsewhere, Mitchell et al. found that enhancing the amount of mesopores surface area in ZSM‐5 had a major impact on retarding deactivation in the methanol to olefin conversion.^14^ Recently, we developed an economical synthesis approach for hierarchical porous ZSM‐5 zeolites by using an ultrasound‐assisted procedure.^15^

Here, we report a novel method for tailoring the pore hierarchy of ZSM‐5 catalysts by changing the crystal size. Unlike the traditional ZSM‐5 zeolite synthesis, we use an MFI‐type silicalite‐1 seeding suspension, on which the Al‐containing ZSM‐5 crystals are grown.^16^ This avoids the need for structure‐directing agents during the crystal growth. The effectiveness of the new catalysts is demonstrated in the dehydration of glycerol to acrolein [Eq. 1]. This benchmark reaction is of general importance,^17^ as a typical acid‐catalyzed dehydration, and is of specific relevance in today's quest for sustainable synthesis of platform chemicals.^18^, ^19^, ^20^ Glycerol is an important biorenewable feedstock. Converting it to acrolein opens routes to bulk chemicals such as acrylic acid, acrylates, acrylonitrile, and acrylamide.^21^, ^22^ The new hierarchical zeolite shows excellent activity and is highly stable. It is at least five times more stable compared with commercial ZSM‐5. Moreover, the fundamental principle of increasing stability by controlling crystal size may apply across the board to zeolite‐catalyzed reactions.
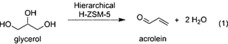



## Results and Discussion

### Synthesis and characterization of ZSM‐5 samples of varied size

To evaluate the effect of ZSM‐5 crystal size on catalytic performance, we prepared four samples of varying crystal sizes. A sample of commercial ZSM‐5 (herein: Com‐ZSM‐5) was compared against three newly synthesized zeolites: microsized crystals (Micro‐ZSM‐5), nanosized crystals (Nano‐ZSM‐5), and nanosized crystals with hierarchical pores (Hier‐ZSM‐5, see the Experimental Section for detailed synthesis procedures). In each case, the concentration of seeding suspension is given after the sample name. For example, Nano‐ZSM‐5‐10 % indicates 10 mol % silica in the seeding suspension, respective to the silica used for crystal growth.

The zeolite crystal size can be precisely controlled by adjusting the amount of added seeding suspension, as shown previously by Xue et al.^23^ In our preliminary tests, the silica concentration in the seeding suspension was varied from 2–15 mol %. Increasing the concentration typically gave many small crystals and vice versa. TEOS is used as the silica source for formation of the silicalite‐1 seeding suspension, and deionized colloidal silica (Ludox TMA) as a highly reactive silica source for ZSM‐5 crystal growth. Remarkably, at low seeding suspension concentrations, we observed the presence of small spherical particles. The high reactivity of the deionized colloidal silica suggests the formation of SiO_2_, which is not incorporated into the ZSM‐5 crystal structure. This was supported by the presence of a broad diffraction peak between 2*θ* values of 18–26°, indicating amorphous SiO_2_. The temperature of the hydrothermal synthesis had a similar effect, in which higher temperatures led to more spherical particles outside of the crystal structure. To avoid the presence of amorphous SiO_2_, Nano‐ZSM‐5 and Hier‐ZSM‐5 were prepared by using at least 10 mol % seeding suspension, 24 h hydrothermal synthesis at 150 °C or higher temperature.

The addition of CTAB has been demonstrated previously by Zhu et al. to introduce mesopores in the zeolite crystals.^24^ They suggested that CTAB is occluded in the crystal structure as a template to form mesopores, and also within the agglomerated crystals to produce cavities of various sizes. This creates hierarchical micro‐/meso‐/macro porosity. We screened several primary alcohols as co‐solvents, aiming at slowing zeolite overgrowth. Ethanol was found to be the best at promoting CTAB inclusion.

Here, we based the synthesis approach for obtaining nanosized crystals on a facile ammonia‐based procedure. A separately prepared suspension of silicalite‐1 zeolite was used as seeding crystals. Preparation of these silicalite‐1 seeds relied on tetrapropylammonium cations (TPA^+^) as the SDA. However, the growth of ZSM‐5 onto the seed was performed in the absence of any SDA. Moreover, ammonia was used directly under hydrothermal conditions to yield zeolites in NH_4_^+^ form. This eliminates the need for time‐consuming ion‐exchange procedures. A further advantage is that this combined approach allows parallel synthesis, enabling quick screening of various catalyst parameters.

The XRD diffractograms for Com‐ZSM‐5, Micro‐ZSM‐5, Nano‐ZSM‐5‐10 %, and Hier‐ZSM‐5‐10 % are shown in Figure S1 in the Supporting Information. These all show characteristic diffraction patterns with typical MFI 2*θ* values between 7.0–9.0°, 12–18°, and 23.0–25.0°. No impurities or amorphous SiO_2_ phases were observed, indicating the formation of pure ZSM‐5 structures. On reducing the crystal size in the synthesized samples, the diffraction peaks became less intense. This may reflect crystal size effects and/or the growth of intercrystallite nanoparticles.

**Figure 2:**
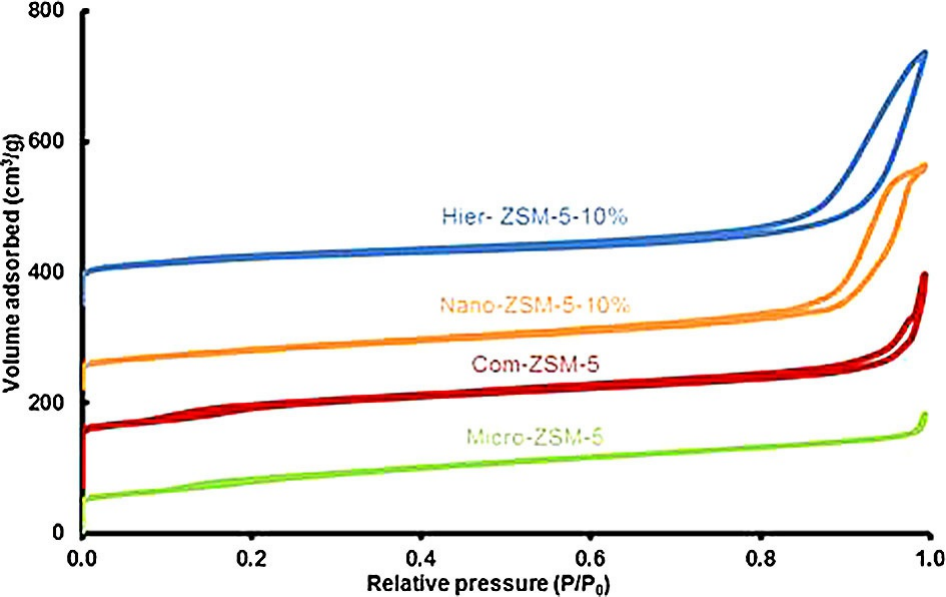
N_2_ physisorption isotherms for ZSM‐5 samples of varied crystal size. Isotherms are stacked with individual offset for clarity.

The nitrogen physisorption isotherms (Figure 2) for the ZSM‐5 samples of varied crystal size show a typical type II curvature.^25^ Note the sharp uptakes at low relative pressure, indicating micropores. The hysteresis loop at low relative pressure (0.1<*P*/*P*_0_<0.2) is related to the effect of fluid‐to‐liquid phase transition of adsorbed nitrogen, on ZSM‐5 zeolites with a high Si/Al ratio.^26^ Nano‐ZSM‐5‐10 % and Hier‐ZSM‐5‐10 % show continuously enhanced desorption at intermediate to high relative pressures (0.45<*P*/*P*_0_<1), without a step down around *P*/*P*_0_=0.45 in the desorption branch. This typical H3 hysteresis loop is ascribed to nitrogen adsorption on the intercrystallite mesopores caused by the agglomeration of nanosized crystals.

Table 1 summarizes the catalysts’ compositions and textural properties. The lowest pore volume was observed for Micro‐ZSM‐5 (*V*_micro_=0.05 cm^3^ g^−1^, *V*_total_=0.26 cm^3^ g^−1^). By reducing the crystal size, Nano‐ZSM‐5‐10 % showed significantly higher pore volume (*V*_micro_=0.07 cm^3^ g^−1^, *V*_total_=0.52 cm^3^ g^−1^). The porosity was increased further for Hier‐ZSM‐5‐10 % (*V*_micro_=0.09 cm^3^ g^−1^, *V*_total_=0.60 cm^3^ g^−1^). For Nano‐ZSM‐5‐10 % and Hier‐ZSM‐5‐10 %, the total pore volumes were higher than for Com‐ZSM‐5 (*V*_micro_=0.12 cm^3^ g^−1^, *V*_total_=0.46 cm^3^ g^−1^), however, the micropore volume was lower. Moreover, BET surface areas are lower for all three synthesized samples than for Com‐ZSM‐5. This supports the XRD findings, which show higher crystallinity for Com‐ZSM‐5 than for the synthesized zeolites. These results show that mesoporosity can be successfully introduced by reducing the crystal size, albeit yielding lower crystallinity for the synthesized samples, as indicated by the lower BET surface areas and micropore volumes.

**Table 1:** Catalyst compositions and textural properties.

Sample	Si/Al [mol mol^−1^]	SA_BET_ [m^2^ g^−1^]	SA_external_ [m^2^ g^−1^]	*V*_micro_ [cm^3^ g^−1^]	*V*_total_ [cm^3^ g^−1^]
Com‐ZSM‐5	63	355	151	0.12	0.46
Micro‐ZSM‐5	57 (63)^[a]^	222	191	0.05	0.26
Nano‐ZSM‐5‐10 %	61 (68)^[a]^	136	136	0.07	0.52
Hier‐ZSM‐5‐10 %	63 (70)^[a]^	255	89	0.09	0.60

[a] Corrected for 10 mol % SiO_2_ in seeding suspension.

**Figure 3:**
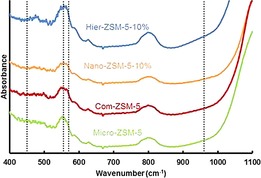
FTIR spectra for ZSM‐5 samples of varied crystal size. Spectra stacked with individual offset for clarity.

The FTIR spectra are shown in Figure 3. All the samples showed a band at 550 cm^−1^ attributed to the asymmetric stretching of the five‐membered ring of MFI zeolite. It is known that the intensity ratio of the band at 550 cm^−1^ relative to the band at 450 cm^−1^ is indicative to the crystallinity by IR.^27^ Remarkably, the Micro‐ZSM‐5 and Hier‐ZSM‐5‐10 % samples show the highest intensity ratio, indicating high crystallinity. Moreover, the MFI‐type framework vibrational band at 550 cm^−1^ appears as a doublet, emphasizing the nanosized properties. Although a broad band at 960 cm^−1^ is observed, its intensity is weak, indicating almost no framework defects or interruptions in the lattice periodicity.^27^, ^28^

The chemical environments of the Al atoms were investigated by using solid‐state ^27^Al magic angle spinning (MAS) NMR spectroscopy. The ^27^Al NMR spectra (Figure S3 in the Supporting Information) show that all samples exhibit a main resonance at a chemical shift of around 56 ppm. This main peak corresponds to tetrahedral coordinated framework Al atoms with Brønsted acidity. No significant signal was observed around 3 ppm. This indicates that no extra‐framework Al was present in these samples, supporting the successful incorporation of solely framework Al.

**Figure 4:**
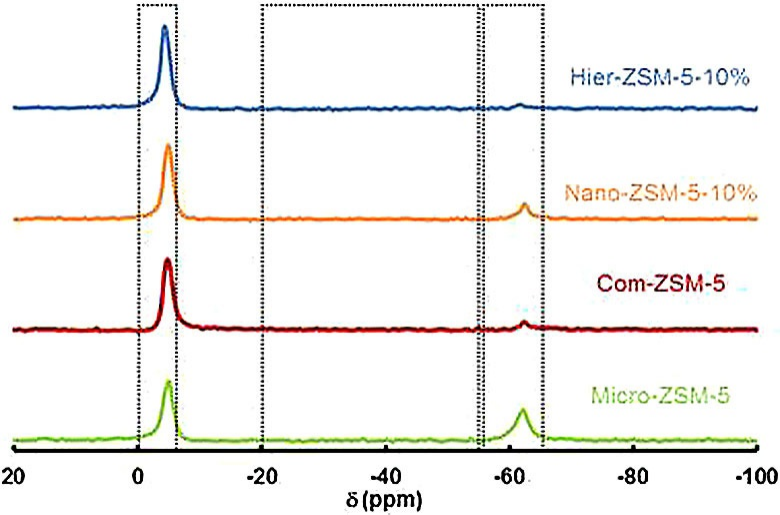
Solid‐state ^31^P MAS NMR spectra by using trimethylphosphine for ZSM‐5 samples of varied crystal size. Spectra stacked with individual offset for clarity.

The nature of the acidic sites was investigated by using solid‐state ^31^P MAS NMR spectroscopy using trimethylphosphine (TMP) as a basic probe molecule (Figure 4). The ^31^P chemical shift at around −55 ppm was assigned to physisorbed TMP. The ^31^P chemical shift in the range 0 to −5 ppm was assigned to TMP chemisorbed on Brønsted acid sites. The ^31^P chemical shift in the range −20 to −55 ppm was assigned to TMP chemisorbed on Lewis acid sites. The quantitative analysis of adsorbed TMP molecules was calculated according to the calibration line established by recording the NMR spectra of standard samples with various adsorbed TMP concentrations. For all catalysts, no Lewis acid chemisorbed probe peak was observed, indicating a purely Brønsted acidic nature of the acid sites (Table 2).

**Table 2:** Chemical properties for ZSM‐5 samples of varied crystal size.

Sample	Peak temperature^[a]^ [°C]		Amount of acid sites^[a]^ [mmol g^−1^]			Brønsted vs. Lewis acid site ratio^[b]^ [%]	Amount of Al^[c]^ [mmol g^−1^]
	Peak I	Peak II	Weak	Strong	Total	Brønsted	
Com‐ZSM‐5	150.6	339.9	0.17	0.16	0.33	100	0.39
Micro‐ZSM‐5	155.4	321.8	0.09	0.11	0.21	100	0.43
Nano‐ZSM‐5‐10 %	156.1	336.6	0.16	0.15	0.31	100	0.41
Hier‐ZSM‐5‐10 %	155.9	323.8	0.12	0.16	0.28	100	0.39

[a] Acidity measured by NH_3_‐TPD. [b] Acidity determined by using a trimethylphosphine probe in ^31^P NMR spectroscopy. [c] Si/Al ratio measured by XRF.

We then used NH_3_‐TPD to quantify the amount and strength of the acid sites. The quantitative results for the ZSM‐5 samples of varied crystal size are presented in Table 2. Herein, the low‐temperature peak (I) was assigned to the weak acid sites, whereas the high‐temperature peak (II) was assigned to the strong ones. The area under the peak provided a quantification of the acid sites. Accordingly, the amount of weak acid sites varied from 0.09 mmol g^−1^ for Micro‐ZSM‐5 to 0.17 mmol g^−1^ for Com‐ZSM‐5. However, the amount of strong acid sites varied less, from 0.11 mmol g^−1^ for Micro‐ZSM‐5 to 0.16 mmol g^−1^ for both Com‐ZSM‐5 and Hier‐ZSM‐5‐10 %. Accordingly, the total amount of acid sites varied from 0.21 mmol g^−1^ for Micro‐ZSM‐5 to 0.33 mmol g^−1^ for Com‐ZSM‐5. Moreover, the total amount of acid sites was compared with the amount of Al, the latter calculated from the Si/Al ratio. The total amount of acid sites was in all cases lower than the amount of Al, indicating that not all, but a large part of the Al atoms contributed to the overall acidity of these ZSM‐5 catalysts.

The Si/Al ratios were determined by X‐ray fluorescence (XRF) after calibration and are shown in Table 1. The values are typically below the nominal Si/Al ratio of 70. However, correcting for the 10 mol % SiO_2_ in the silicalite‐1 seeds shows that the Si/Al ratio in the actual Al‐containing ZSM‐5 material is 68 % for Nano‐ZSM‐5‐10 % and 70 % for Hier‐ZSM‐5‐10 %. This demonstrates that Al can be quantitatively incorporated by using the novel seed‐induced synthesis approach.

### Structure and morphology of ZSM‐5 samples with varied crystal size

**Figure 5:**
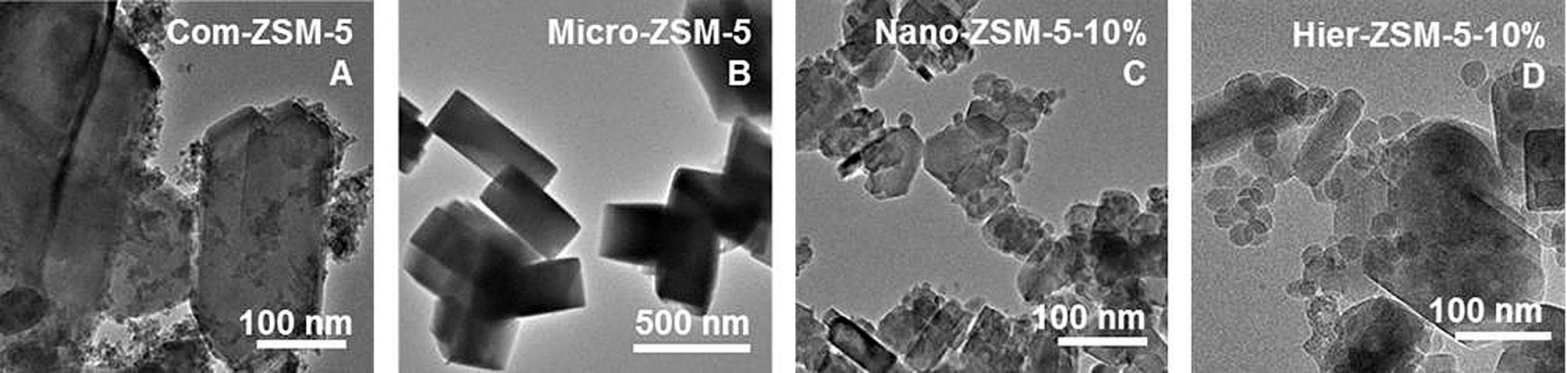
TEM images for A) Com‐ZSM‐5, B) Micro‐ZSM‐5, C) Nano‐ZSM‐5‐10 %, and D) Hier‐ZSM‐5‐10 %.

**Figure 6:**
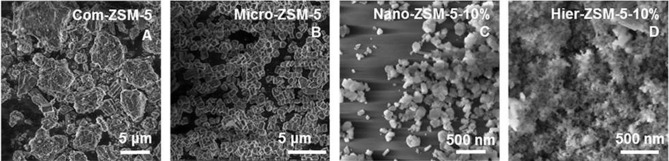
SEM images for A) Com‐ZSM‐5, B) Micro‐ZSM‐5, C) Nano‐ZSM‐5‐10 %, and D) Hier‐ZSM‐5‐10 %.

The crystal size and morphology were analyzed by TEM (Figure 5) and SEM (Figure 6). The TEM image of Com‐ZSM‐5 (Figure 5 A) shows a broad crystal size distribution. Micron‐sized crystals were observed with agglomerated sub‐micron crystals. This was confirmed by the SEM image (Figure 6 A), which shows rough micron‐sized crystals, with presence of sub‐micron crystals on the surface. These results agree with the high BET surface area, high micropore volume, and limited mesopores volume, as shown by N_2_ physisorption. The TEM and SEM images of Micro‐ZSM‐5 (Figure 5 B, Figure 6 B) showed a highly uniform crystal morphology and the narrow crystal surface was typically smooth, which explained the low total pore volume as observed by N_2_ physisorption. The TEM and SEM images of Nano‐ZSM‐5‐10 % (Figure 5 C, Figure 6 C) showed the formation of nanosized crystals, with around 160 nm average crystal size. A narrow distribution in shape and size was observed, with crystals clearly showing the typical elongated hexagonal shape of ZSM‐5. The TEM and SEM images of Hier‐ZSM‐5‐10 % (Figure 5 D, Figure 6 D) showed a bimodal crystal size distribution. ZSM‐5 crystals of around 150 nm average size were observed, together with very small crystals of around 25 nm average size. The smaller crystals are overgrown on top of the larger ZSM‐5 crystals. Remarkably, no amorphous SiO_2_ phase was observed by XRD, indicating that these smaller crystals are grown in MFI morphology. For the synthesis of Hier‐ZSM‐5‐10 %, EtOH was used to retard crystal growth. These results suggest that the overgrowth can be reduced, to further increase porosity.

### Catalyst testing of ZSM‐5 samples with varied crystal size

The catalytic performance of the ZSM‐5 samples of varied crystal size was tested in the dehydration of glycerol to acrolein. Various solid catalysts have been reported for this reaction, including zeolites, metal pyrophosphates, Nb_2_O_5_/SiO_2_, and heteropolyacids.^29^, ^30^, ^31^ Liu and co‐workers obtained 96 % conversion with 83 % selectivity towards acrolein by using a Nd_4_(P_2_O_7_)_3_ catalyst.^32^ We previously showed that using Nb_2_O_5_/SiO_2_ catalysts, glycerol conversion and selectivity to acrolein can be controlled by the niobium loading and calcination temperature.^33^ Elsewhere, de Oliveira and co‐ workers studied liquid‐phase glycerol dehydration by using zeolite catalysts.^28^ They found that catalytic activity was not directly correlated to the Si/Al ratio. Instead, the catalyst structure, porosity, and acid site strength were the key factors. By using the selectivity to acrolein after 10 h at 250 °C, we recently demonstrated the importance of mesoporosity and suitable acidity for the high activity and long lifetime of ZSM‐5.^34^

**Figure 7:**
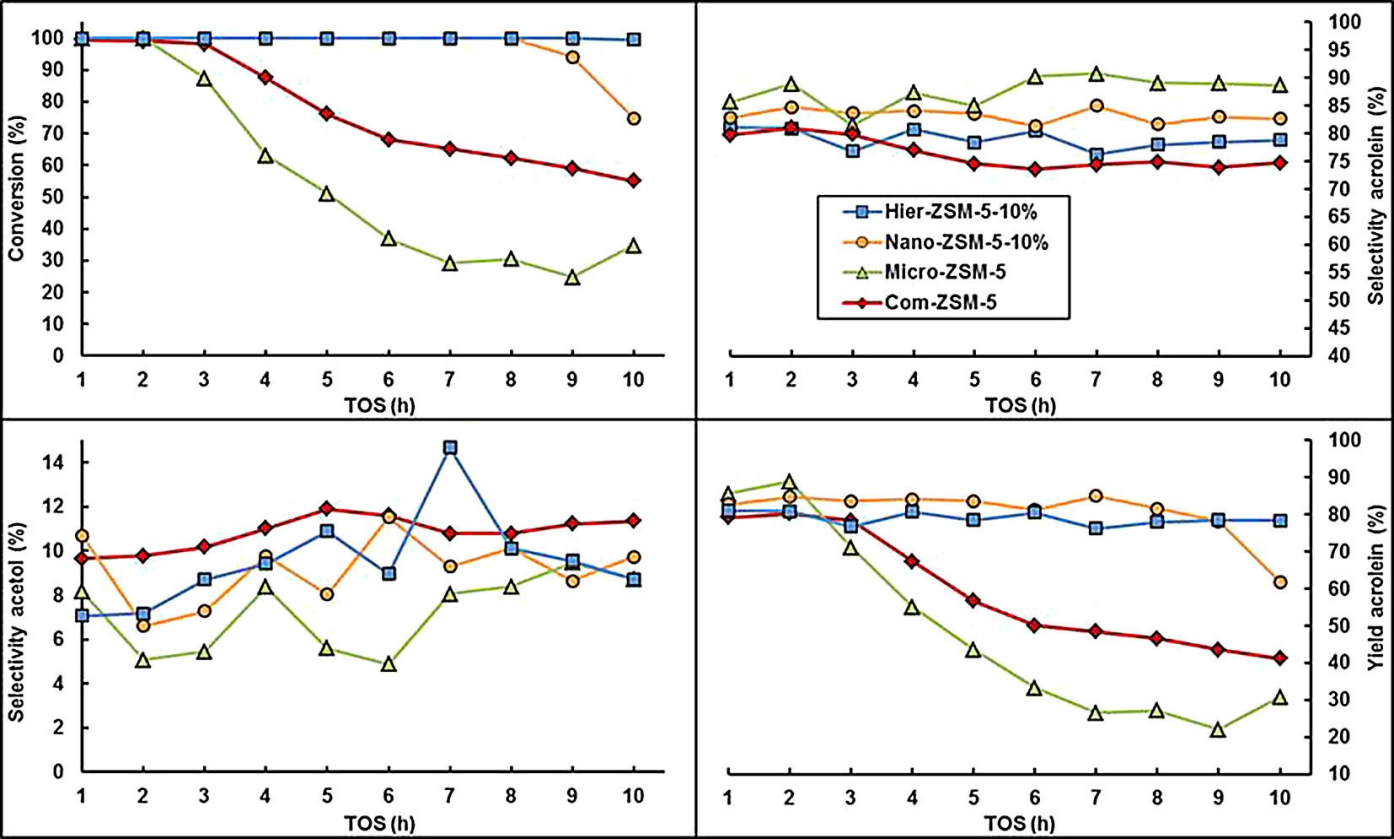
Catalytic results for glycerol conversion, acrolein and acetol selectivities, and acrolein yield for Com‐ZSM‐5 (red diamonds), Micro‐ZSM‐5 (green triangles), Nano‐ZSM‐5‐10 % (orange squares), and Hier‐ZSM‐5‐10 % (blue circles).

In a typical reaction, the catalytic activity was studied by using a 20 wt % aqueous glycerol feed. The reaction was performed at 320 °C, at a WHSV of 2.4 h^−1^ (see the Experimental Section for full details). Acrolein and hydroxyacetone, also known as acetol, are generally considered the main products.^30^, ^31^ In our experiments, acrolein and acetol typically gave >90 % of combined selectivity. Hourly samples were taken and analyzed by gas chromatography. The reaction profiles in Figure 7 show the conversion of glycerol, the selectivity to acrolein and acetol, and the overall yield of acrolein.

The Com‐ZSM‐5 catalyst gave full conversion for 2 h on stream, before deactivating rapidly to 41 % after 10 h. Its selectivity towards acrolein was around 81 % after 2 h and decreased to 75 % after 10 h. The Micro‐ZSM‐5 sample showed the most rapid deactivation, after 2 h on stream. However, the highest selectivity towards acrolein was observed for Micro‐ZSM‐5 (89 % after 10 h). The Nano‐ZSM‐5‐10 % sample showed high stability, retaining full conversion for up to 8 h on stream. The selectivity to acrolein remained above 83 % after 10 h on stream. Hier‐ZSM‐5‐10 % showed the best stability, demonstrating 99 % conversion after 10 h on stream. However, acrolein selectivity for Hier‐ZSM‐5‐10 % was significantly lower than for Micro‐ZSM‐5 and Nano‐ZSM‐5‐10 %. The selectivity towards acetol was lower for all synthesized samples than for Com‐ZSM‐5. The acrolein yields after 10 h on stream for Nano‐ZSM‐5‐10 % and Hier‐ZSM‐5‐10 % were 62 % and 78 %, respectively. This is a remarkable increase over the 41 % yield observed for Com‐ZSM‐5.

The highest BET surface area and micropore volume were observed for Com‐ZSM‐5. However, its catalytic stability was significantly lower than for Nano‐ZSM‐5‐10 % and Hier‐ZSM‐5‐10 %. The low mesopore volume of Com‐ZSM‐5, as demonstrated by nitrogen physisorption, is likely the cause of the low activity. This loss of activity is typical for coking, which blocks the pores, denying accessibility to acidic sites. Similarly, Micro‐ZSM‐5 contained more acid sites than Nano‐ ZSM‐5‐10 %, but was found to be less active. This may reflect its low pore volume and poor resistance to coking. The high activity of Nano‐ZSM‐5‐10 % is likely the direct result of the high number of weak acid sites combined with the large mesoporosity. The selectivity towards acrolein was the highest for the Micro‐ZSM‐5 sample. The XRD results of Micro‐ZSM‐5 suggested higher crystallinity than for Nano‐ZSM‐5‐10 % and Hier‐ZSM‐5‐10 %. Moreover, TEM and SEM showed a highly uniform shape for Micro‐ZSM‐5. High crystallinity and crystal shape and size uniformity proved crucial to obtain high acrolein selectivity. Thus, we focused next on optimizing the hydrothermal synthesis conditions for Nano‐ZSM‐5 and Hier‐ZSM‐5.

### Structural optimization of ZSM‐5 zeolites

To reduce crystal overgrowth for Nano‐ZSM‐5, preliminary tests showed that using 15 mol % seeding suspension and 30 h of hydrothermal synthesis time at 150 °C were optimal conditions to incorporate Si into uniformly sized ZSM‐5 crystals. However, for Hier‐ZSM‐5, with 15 mol % seeding suspension, after 30 h hydrothermal synthesis at 150 °C, overgrowth of small crystals was still observed. With the addition of CTAB and EtOH, the hydrothermal synthesis for 30 h at 150 °C proved to be too short to fully incorporate Si into a uniformly sized ZSM‐5 phase. To optimize crystallinity and porosity, a series of Hier‐ZSM‐5‐15 % samples was prepared with 30–60 h of hydrothermal synthesis time at 150 °C. In each case, the hydrothermal synthesis time is given after the sample name. For example, Hier‐ZSM‐5‐15 %‐30 h indicates 30 h hydrothermal synthesis at 150 °C.

The XRD diffractograms for Nano‐ZSM‐5‐15 % synthesized after 30 h at 150 °C and Hier‐ZSM‐5‐15 % synthesized after 30, 40, 50, and 60 h at 150 °C are shown in Figure S2 (in the Supporting Information). The characteristic MFI structure diffraction patterns are observed between 7.0–9.0°, 12–18°, and 23.0–25.0° 2*θ*. No impurities or amorphous SiO_2_ phases were observed. Sharp and narrow peaks were observed for Nano‐ZSM‐5‐15 %, indicating highly crystalline ZSM‐5 material. For Hier‐ZSM‐5‐15 %, the sharpest and most narrow peaks were observed for the sample prepared after 50 h hydrothermal synthesis time.

**Figure 8:**
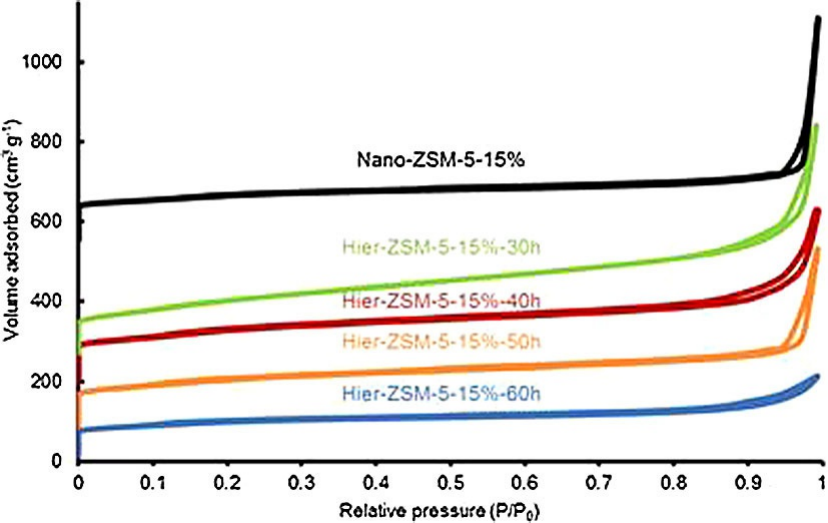
N_2_ physisorption isotherms for ZSM‐5 samples of optimized synthesis conditions. Isotherms are stacked with individual offset for clarity.

The nitrogen physisorption isotherms (Figure 8) of the optimized series of ZSM‐5 samples show typical type II curvature. The sharp uptakes at low relative pressure indicate the presence of micropores. Nano‐ZSM‐5‐10 % and Hier‐ZSM‐5‐10 % show continuously enhanced desorption at intermediate to high relative pressures (0.45<*P*/*P*_0_<1), without a step down around *P*/*P*_0_=0.45 in the desorption branch.

The compositions and textural properties of the optimized ZSM‐5 samples are summarized in Table 3. For all optimized ZSM‐5 samples, BET surface areas above 350 m^2^ g^−1^ were observed. For Hier‐ZSM‐5‐15 %, the BET surface area decreases from 483 m^2^ g^−1^ after 30 h of hydrothermal synthesis time, down to 350 m^2^ g^−1^ after 60 h. These results indicate that more Si is incorporated into single‐phase ZSM‐5 crystals, and thereby crystallinity is increased. This is further supported by the observation that the external surface area decreases at longer hydrothermal synthesis times (from 349 m^2^ g^−1^ down to 70 m^2^ g^−1^), whereas the internal surface area increases (from 134 m^2^ g^−1^ up to 280 m^2^ g^−1^). Moreover, with increased hydrothermal synthesis time, the micropore volume increases (from 0.06 cm^3^ g^−1^ up to 0.13 cm^3^ g^−1^), whereas the total pore volume decreases (from 0.84 cm^3^ g^−1^ down to 0.33 cm^3^ g^−1^). However, the micropore volume did not decrease after 50 h, whereas the total pore volume did decrease after 50 h. This indicates that for Hier‐ZSM‐5‐15 %, the optimal crystallinity and porosity is obtained after 50 h. These results are in agreement with the XRD results, in which Hier‐ZSM‐5‐15 %‐50 h showed the highest crystallinity.

**Table 3:** Compositions and textural properties for ZSM‐5 samples of optimized synthesis conditions.

Sample	Si/Al [mol mol^−1^]	SA_BET_ [m^2^ g^−1^]	SA_external_ [m^2^ g^−1^]	*V*_micro_ [cm^3^ g^−1^]	*V*_total_ [cm^3^ g^−1^]
Nano‐ZSM‐5‐15 %	60	368	109	0.12	0.82
Hier‐ZSM‐5‐15 %‐30 h	59 (69)^[a]^	483	349	0.06	0.84
Hier‐ZSM‐5‐15 %‐40 h	60 (70)^[a]^	389	183	0.12	0.66
Hier‐ZSM‐5‐15 %‐50 h	63 (74)^[a]^	390	150	0.13	0.70
Hier‐ZSM‐5‐15 %‐60 h	62 (73)^[a]^	350	70	0.13	0.33

[a] Corrected for 15 mol % SiO_2_ in seeding suspension.

Furthermore, to investigate the stability of the hierarchical structure, ultrasonic treatment was applied for the Hier‐ZSM‐5‐15 % samples. The physical properties of these samples are presented in Table S1 (in the Supporting Information), whereas the XRD diffractograms are shown in Figure S3. These results show that even after the ultrasonic treatment under harsh conditions, the samples retain most of their crystallinity and hierarchical porosity.

**Figure 9:**
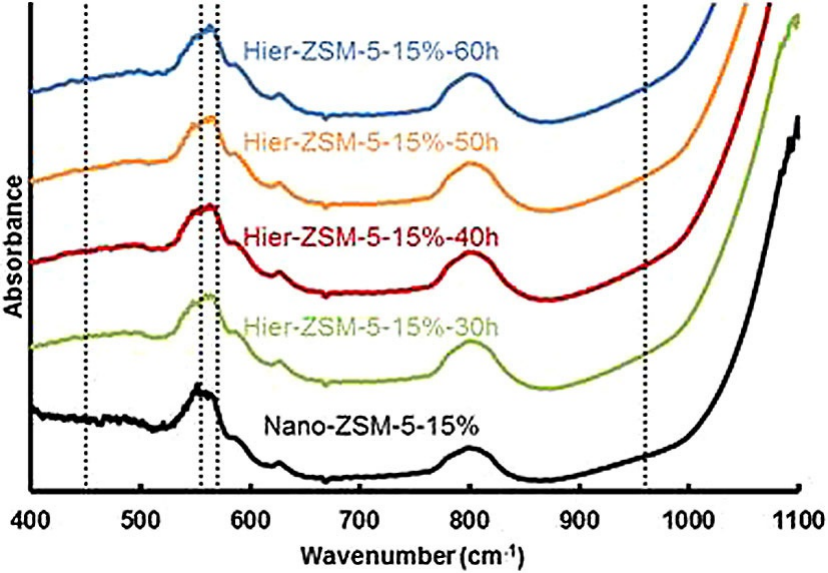
FTIR spectra for ZSM‐5 samples of optimized synthesis conditions. Spectra stacked with individual offset for clarity.

The FTIR spectra are shown in Figure 9. For all samples, a strong doublet band at 550 cm^−1^ was observed, whereas almost no band is observed at 450 cm^−1^, indicating highly crystalline MFI‐type structures.^27^ Moreover, the broad band observed at 960 cm^−1^ was weak, indicating high crystallinity for these nanosized and hierarchical ZSM‐5 catalysts.^27^

The ^27^Al NMR spectra (Figure S4 in the Supporting Information) show that all samples exhibit a main resonance at approximately 56 ppm. This main peak corresponds to tetrahedral coordinated framework Al atoms with Brønsted acidity, indicating the successful incorporation of only framework Al. The ^29^Si NMR spectra (Figure S5) show that all catalysts exhibit resonance at two chemical shift positions. The first peak around −112 ppm corresponds to Si(OSi)_4_ (Q^4^) species, whereas the second peak around −103 ppm indicates (AlO)–Si(OSi)_3_ (Q^3^) species. The intense Q^3^ peak is attributed to the incorporation of Al and/or the presence of silanol groups. The large amount of silanol groups likely comes from the mesoporous surface area. Moreover, no other bands could be observed, indicating the high crystallinity of these samples.

**Figure 10:**
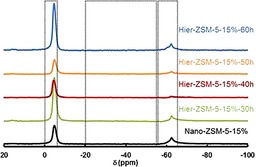
Solid‐state ^31^P MAS NMR spectra by using trimethylphosphine for ZSM‐5 samples of optimized synthesis conditions. Spectra stacked with individual offset for clarity.

The nature of the acidic sites was investigated by using solid‐state ^31^P MAS NMR spectroscopy using trimethylphosphine as a basic probe molecule (Figure 10). All samples showed Brønsted acidity only (Table 4).

**Table 4:** Chemical properties for ZSM‐5 samples of optimized synthesis conditions.

Sample	Peak temperature [°C]		Amount of acid sites^[a]^ [mmol g^−1^]			Brønsted vs. Lewis acid site ratio^[b]^ [%]	Amount of Al^[c]^ [mmol g^−1^]
	Peak I	Peak II	Weak	Strong	Total	Brønsted	
Nano‐ZSM‐5‐15 %	161.6	343.5	0.15	0.22	0.37	100	0.41
Hier‐ZSM‐5‐15 %‐30 h	168.9	346.2	0.13	0.19	0.32	100	0.42
Hier‐ZSM‐5‐15 %‐40 h	159.8	340.1	0.15	0.21	0.37	100	0.41
Hier‐ZSM‐5‐15 %‐50 h	163.5	344.2	0.15	0.23	0.39	100	0.39
Hier‐ZSM‐5‐15 %‐60 h	161.6	340.6	0.16	0.24	0.39	100	0.40

[a] Acidity measured by NH_3_‐TPD. [b] Acidity determined by using a trimethylphosphine probe in ^31^P NMR spectroscopy. [c] Si/Al ratio measured by XRF.

The amount and strength of the acid sites for the optimized ZSM‐5 samples, as determined by NH_3_‐TPD, are shown in Table 4. Moreover, the amount of Al is shown, as calculated based on the Si/Al ratio. For Nano‐ZSM‐5‐15 %, the amount of Al (0.41 mmol g^−1^) contributed nearly quantitatively to the total amount of acid sites (0.37 mmol g^−1^). For Hier‐ZSM‐5‐15 %, both the amounts of weak and strong acid sites increased with longer hydrothermal synthesis time. The total amount of acid sites increased from 0.32 mmol g^−1^ after 30 h, up to 0.39 mmol g^−1^ after 60 h. After 50 h of hydrothermal synthesis time, the total amount of Al (0.39 mmol g^−1^) contributed quantitatively to the amount of acid sites. This very high acidity proves the effectiveness of the direct ammonia‐based synthesis approach.

Finally, the Si/Al ratios of the optimized ZSM‐5 samples vary between 59–63. Considering that 15 mol % of SiO_2_ was in the silicalite‐1 seeds and thus 85 mol % of SiO_2_ in the ZSM‐5 phase, the actual Si/Al ratio in the ZSM‐5 phase varied between 69–74. This shows that Al is fully incorporated into the ZSM‐5 phase.

### Structure and morphology of ZSM‐5 samples with optimized synthesis conditions

**Figure 11:**
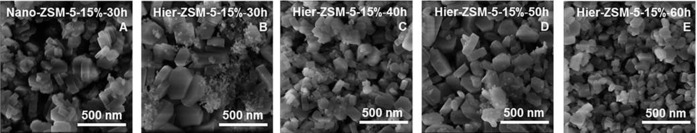
TEM images for A) Nano‐ZSM‐5‐15 %, B) Hier‐ZSM‐5‐15 %‐30 h, C) Hier‐ZSM‐5‐15 %‐40 h, D) Hier‐ZSM‐5‐15 %‐50 h, and E) Hier‐ZSM‐5‐15 %‐60 h.

**Figure 12:**
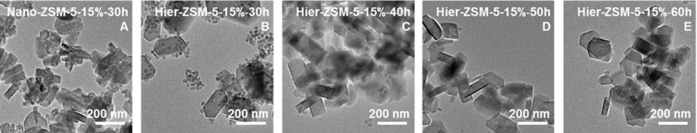
SEM images for A) Nano‐ZSM‐5‐15 %, B) Hier‐ZSM‐5‐15 %‐30 h, C) Hier‐ZSM‐5‐15 %‐40 h, D) Hier‐ZSM‐5‐15 %‐50 h, and E) Hier‐ZSM‐5‐15 %‐60 h.

Crystal size and morphology were analyzed by TEM (Figure 11) and SEM (Figure 12). For Nano‐ZSM‐5‐15 % synthesized after 30 h of hydrothermal synthesis time at 150 °C, only minor crystal overgrowth was observed by TEM (Figure 11 A) and SEM (Figure 12 A). The average crystal size was around 150 nm. For Hier‐ZSM‐5‐15 %, after hydrothermal synthesis at 150 °C, crystal overgrowth was clearly visible after 30 h. By increasing the hydrothermal synthesis time up to 60 h, the amount of overgrowth crystals was nearly completely reduced (Figure 12 E). The average crystal size was around 150 nm. Optimizing the synthesis conditions for Nano‐ZSM‐5 and Hier‐ZSM‐5, by using 15 mol % SiO_2_ seeding suspension for hydrothermal synthesis at 150 °C, significantly improved the incorporation of Si into uniformly sized ZSM‐5 crystals.

### Catalytic testing of ZSM‐5 samples prepared under optimized conditions

**Figure 13:**
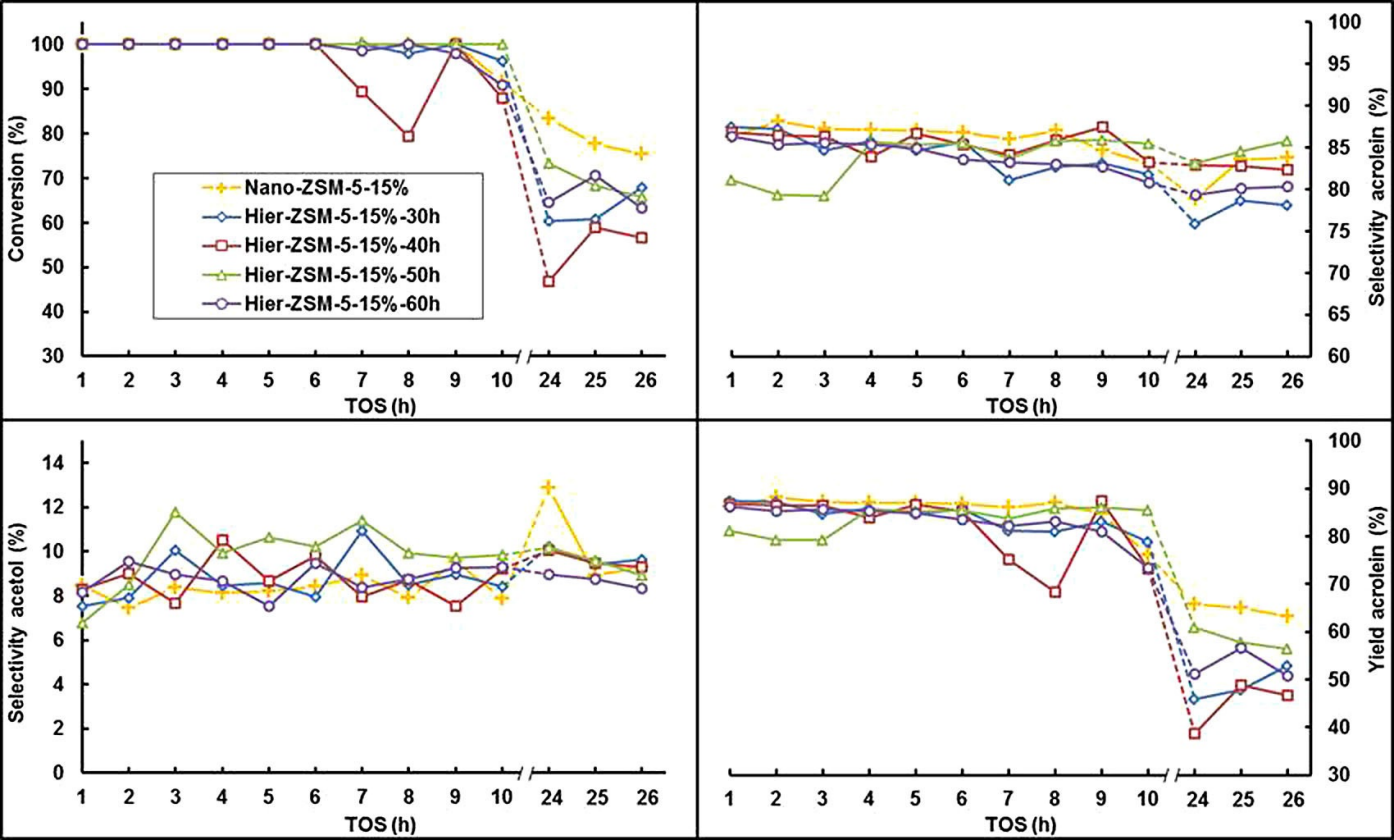
Catalytic results for glycerol conversion, selectivities towards acrolein and acetol, and yield of acrolein. Measurements between 10–24 h have been omitted for clarity, indicated by the dashed lines. Nano‐ZSM‐5‐15 % (yellow crosses), Hier‐ZSM‐5‐15 %‐30 h (blue diamonds), Hier‐ZSM‐5‐15 %‐40 h (red squares), Hier‐ZSM‐5‐15 %‐50 h (green triangles), Hier‐ZSM‐5‐15 %‐60 h (purple circles).

The catalytic reaction profiles for the ZSM‐5 samples with optimized synthesis conditions are shown in Figure 13. Measurements between 10–24 h have been omitted for clarity, indicated by the dashed lines. Nano‐ZSM‐5‐15 % showed 91 % conversion after 10 h on stream, with 83 % acrolein selectivity. This is a significant improvement over Nano‐ZSM‐5‐10 %, which attained 75 % conversion with 83 % acrolein selectivity after 10 h. Furthermore, Nano‐ZSM‐5‐15 % showed moderated decay in stability, and 76 % conversion and 84 % acrolein selectivity are obtained after 26 h. Finally, 63 % acrolein yield was obtained after 26 h. For Hier‐ZSM‐5‐15 %, conversions after 10 h ranged from 88 % for Hier‐ZSM‐5‐15 %‐40 h, to 100 % conversion for Hier‐ZSM‐5‐15 %‐50 h. Moreover, acrolein selectivity after 10 h ranged from 81 % for Hier‐ZSM‐5‐15 %‐60 h to 85 % for Hier‐ZSM‐5‐15 %‐50 h. After 26 h, conversion ranged from 57 % for Hier‐ZSM‐5‐15 %‐40 h to 68 % for Hier‐ZSM‐5‐15 %‐30 h. Furthermore, acrolein selectivity after 26 h ranged from 78 % (Hier‐ZSM‐5‐15 %‐30 h) to 86 % (Hier‐ZSM‐5‐15 %‐50 h). For the hierarchical ZSM‐5 series prepared with 15 mol % seeding suspension, all samples showed increased acrolein selectivity after 10 h compared with Hier‐ZSM‐5‐10 %. Moreover, the highest acrolein yields were obtained with Hier‐ZSM‐5‐15 %‐50 h, both after 10 h (85 % yield) and also after 26 h (56 % yield).

Of the five optimized samples, Nano‐ZSM‐5‐15 % showed the best performance. The high surface area and total pore volume may be responsible for its high activity and stability. Moreover, the uniform shape and size of the Nano‐ZSM‐5‐15 % crystals are likely responsible for the high selectivity towards acrolein. For the series of Hier‐ZSM‐5‐15 % samples, the largest amounts of micropore volume, and the highest acrolein yields were found for 30 h and 50 h of hydrothermal synthesis time. This supports the indication that mesopore volume is crucial for stability. Moreover, a very high amount of weak acid sites and complete contribution of Al atoms as acid sites was observed for ZSM‐5‐15 %‐50 h, and the highest acrolein selectivity after 26 h was also observed for this sample. This confirms that the number of weak acid sites is crucial for high selectivity towards acrolein.

## Conclusions

Nanosized ZSM‐5 materials were successfully prepared by growing the crystals onto pre‐prepared silicalite‐1 seeds. Crystal sizes are easily controlled by adjusting the concentration of seeding suspension. The deionized colloidal silica was reacted directly with ammonia, eliminating the time‐consuming ion‐exchange step. By using the silicalite‐1 seeds, ZSM‐5 crystals could be grown under controlled conditions, without any structure‐directing agent. After optimization, this approach allowed for the facile synthesis of ZSM‐5 crystals with hierarchical structures of micro‐/meso‐/macro pores. The structural properties of the materials were tailored during the initial hydrothermal synthetic step. The clustered hierarchical morphology was shown to be a result of the initial synthesis procedure, prior to drying and calcination. Hence, reducing the crystal size leads directly to crystal agglomeration.

The nanosized ZSM‐5 samples were then tested in the catalytic dehydration of glycerol to acrolein, and compared against commercial ZSM‐5. The new zeolites exhibit a five‐fold improvement in catalytic stability with time on stream. Moreover, the overall acrolein yield after 10 h more than doubled. The improved stability is attributed to enhanced resistance to coking thanks to the hierarchical structure of micropores, mesopores, and macropores. Overall, these highly active and selective catalysts demonstrate the importance of nanoscale zeolite design. The generality of this method and its ease opens opportunities for applications across the board in hierarchical zeolite synthesis and catalysis.

## Experimental Section

### Materials and instrumentation

Powder X‐ray diffraction (XRD) patterns were recorded with a Bruker D8 Advance X‐ray diffractometer (40 kV, 40 mA) with CuK_α_ radiation (*λ*=0.15405 nm), equipped with a LynxEye detector. Transmission electron microscopy (TEM) images were recorded with a G2 20‐s‐twin microscope (Tecnai) operated at 200 kV. Samples were dispersed in ethanol and then applied onto carbon films supported on copper grids. Scanning electron microscopy (SEM) images were recorded with a Nova Nano SEM 450 (FEI) microscope operated at 2–3 kV. Average crystal diameters were measured over the longitudinal hexagonal axis. The zeolite Si/Al ratios and Al amount per gram of catalysts were determined by X‐ray fluorescence (XRF) with a Bruker‐AXS spectrometer. Nitrogen physisorption measurements were performed with a Quantachrome Autosorb‐iQ apparatus, at −195.79 °C. Samples were evacuated prior to measurements at 250 °C for 3 h under dynamic vacuum. The surface areas (SA_BET_) were calculated by using the Brunauer–Emmett–Teller (BET) method. The external surface areas (SA_external_) and the micropore volumes (*V*_micro_) were calculated by using the t‐method. The pore diameter and distributions were calculated by using the Barret–Joyner–Halenda (BJH) method, applying the adsorption branch. The single point total pore volumes (*V*_total_) were determined at *P*/*P*_0_=0.99. The amount and strengths of acid groups were determined by ammonia temperature‐programmed desorption (NH_3_‐TPD) by using a Micromeritics AutoChem 2920 analyzer. Each catalyst sample (0.1 g) was heated to 550 °C in a helium flow for 3 h and then cooled to 80 °C. NH_3_ adsorption was performed under a flow of 10 vol % NH_3_/He (30 mL min^−1^) for 1 h. The NH_3_‐TPD was promptly started at a heating rate of 10 °C min^−1^ from 80 to 600 °C. FTIR spectroscopy was performed by using a Nicolet FTIR 6700 spectrometer. Framework vibrational bands of the ZSM‐5 zeolites were obtained with a KBr tablet at room temperature.

Solid‐state ^27^Al magic‐angle spinning (MAS) NMR spectra were recorded by using a Bruker DSX 300 spectrometer at 12 KHz spinning rate. The operating frequency was 104.3 MHz, 18° pulse, and recycle delay of 2 s. Solid‐state ^29^Si magic‐angle spinning (MAS) NMR spectra were recorded by using a Bruker DSX 300 spectrometer at 4 KHz spinning rate. The operating frequency was 79.6 MHz, 60° pulse, and recycle delay of 120 s. Trimethylphosphine (TMP) was used as a basic probe molecule to characterize Brønsted and Lewis acidity through NMR spectroscopy. Solid‐state ^31^P magic‐angle spinning (MAS) NMR spectra were recorded by using an AVANCE III 400WB spectrometer at 12 KHz spinning rate. The operating frequency was 162.1 MHz, 30° pulse, and recycle delay of 15 s. A certain amount of catalyst was placed in a home‐made glass tube with a constriction fitting precisely into a double bearing 7 mm Bruker zirconia rotor. The glass tube was connected to a vacuum system. After pre‐treating the catalyst at 200 °C for 2 h under vacuum (101 Pa), the NMR tube was immersed in liquid nitrogen to introduce a specific amount of adsorbed TMP molecules with a molar ratio of 0.6 to the catalyst. The ^31^P chemical shift at around −55 ppm was assigned to physisorbed TMP. The ^31^P chemical shift in the range 0 to −5 ppm was assigned to TMP chemisorbed on Brønsted acid sites. The ^31^P chemical shift in the range −20 to −55 ppm was assigned to TMP chemisorbed on Lewis acid sites. The quantitative analysis of adsorbed TMP molecules was calculated according to the calibration line established by recording the NMR spectra of standard samples with various adsorbed TMP concentrations.

Unless stated otherwise, chemicals were purchased from commercial sources and used as received. Commercial ZSM‐5 in the protonic form with Si/Al ratio of 70 was obtained from the Nankai University Catalyst Plant. Prior to use, all glassware, Teflon‐lined reactors, and ceramics were rinsed with deionized water and dried, to avoid the presence of alkali counter‐ions. All catalysts were synthesized by using the same batch of silicalite‐1 seeding suspension, which was prepared separately beforehand. The subsequent one‐pot synthesis procedures may be performed entirely in parallel, including hydrothermal synthesis, drying, and calcination. As shown previously, the crystal size can be precisely controlled by the amount of added seeding suspension.^23^ Hier‐ZSM‐5‐15 % zeolites were treated by ultrasonication for 20 min in deionized water by using a ultrasonic processor (Shanghaizhixin Co., 60 % amplitude, 1 cycle, and 50 Hz).

### Procedure for preparing Micro‐ZSM‐5

This is an modification to a published procedure.^35^ NaOH (0.266 g, 6.65 mmol), NaAlO_2_ (0.160 g, 1.95 mmol), PEG‐20 000 (4 g, 0.2 mmol), and tetrapropylammonium hydroxide (TPAOH; 25 wt %, 43.2 g, 53.1 mmol) were mixed in a Teflon‐lined reactor and tetraethyl orthosilicate (TEOS; 28 g, 134.4 mmol) was then added, and the mixture was stirred for 2 h and then dried overnight at 60 °C. Deionized water (5 mL) was added to the dried gel, and the vessel was placed in a stainless‐steel autoclave for hydrothermal synthesis at 160 °C. After 12 h, the solid was filtered and washed several times with deionized water, giving white needle crystals. The crystals were dried overnight at room temperature. The sample was calcined in air, first at 300 °C for 7 h to remove the SDA and subsequently at 550 °C for 6 h (5 °C min^−1^ ramp rate). After calcination, the sample was ion‐exchanged three times with 1 m NH_4_NO_3(aq)_ (20 mL g^−1^) at 80 °C for 3 h to remove organic impurities and exchange the zeolite from the Na^+^ form to the NH_4_^+^ form. The protonic H^+^‐ZSM‐5 zeolite form was obtained in a muffle furnace by calcination in static air at 550 °C for 7 h (2 °C min^−1^ ramp rate).

### Procedure for preparing the silicalite‐1 seeding suspension

TEOS (41.67 grams, 0.2 mol SiO_2_) TPAOH(aq) (50 wt %, 29.17 g, 71.72 mmol), and deionized water (29.17 mL) were mixed in a Teflon‐lined vessel. The mixture was stirred for 24 h at room temperature to hydrolyze the TEOS. Subsequently, the vessel was transferred to a stainless‐steel autoclave, which was heated at 80 °C for 72 h. The resulting seeding suspension was used directly without further filtration or washing. The seeding suspension could be stored in a closed glass container up to one week at 4 °C prior to use.

### Procedure for preparing Nano‐ZSM‐5 and Hier‐ZSM‐5

Example 1: Nano‐ZSM‐5‐10 % and Hier‐ZSM‐5‐10 %. The synthesis mixtures were prepared in a Teflon‐lined reactor. Al_2_(SO_4_)_3_–18 H_2_O (476 mg, 0.71 mmol) was dissolved in deionized water (40 mL). An ammonia solution (25–28 wt % NH_4_OH, 15 mL) was added and the mixture was stirred for several minutes. Then, deionized colloidal silica (Ludox TMA, 17.65 g, 0.10 mol SiO_2_) was added, followed by adding the seeding suspension (5.0 g, 10 mmol SiO_2_). For Nano‐ZSM‐5‐10 %, the reaction mixture was ready for hydrothermal treatment. However, for Hier‐ZSM‐5‐10 %, cetyltrimethylammonium bromide (CTAB, 765 mg, 2.09 mmol) and ethanol (6 mL) were added directly after addition of the seeding suspension. The mixtures were stirred for several minutes, before transferring the reactors to stainless‐steel autoclaves. Nano‐ZSM‐5‐10 % was heated to 175 °C for 24 h. Hier‐ZSM‐5‐10 % was heated to 175 °C for 41 h.

Example 2: Nano‐ZSM‐5‐15 % and Hier‐ZSM‐5‐15 %. The synthesis mixtures were prepared in a Teflon‐lined reactor. Al_2_(SO_4_)_3_ **⋅**18 H_2_O (476 mg, 0.71 mmol) was dissolved in deionized water (40 mL). After that, ammonia solution (25–28 wt % NH_4_OH, 15 mL) was added and the mixture was stirred for several minutes. Then, deionized colloidal silica (Ludox TMA, 17.65 g, 0.10 mol SiO_2_) was added, followed by adding the seeding suspension (7.5 g, 15 mmol SiO_2_). For Nano‐ZSM‐5‐15 %, the reaction mixture was ready for hydrothermal treatment. However, for Hier‐ZSM‐5‐15 %, cetyltrimethylammonium bromide (CTAB, 765 mg, 2.09 mmol) and ethanol (6 mL) were added directly after addition of the seeding suspension. The mixtures were stirred for several minutes, before transferring the reactors to stainless‐steel autoclaves. Nano‐ZSM‐5‐15 % was heated to 150 °C for 30 h. Hier‐ZSM‐5‐10 % was heated to 150 °C for 30–60 h.

After the hydrothermal synthesis, a white powder was collected with 40 mL of additional deionized water. The solid was separated by centrifuging at 8000 rpm for 20 min. After decanting the mother liquid, the pellets were broken inside the centrifugation vial and re‐suspended in 60–80 mL of deionized water, to wash the catalysts three times. The solids were dried at 110 °C overnight. The protonic zeolite forms were obtained by calcination in a muffle furnace in static air, at 550 °C for 7 h (ramp rate 2 °C min^−1^). No ion‐exchange procedure was necessary and the catalysts were used directly after calcination.

### Procedure for catalytic testing

The gas‐phase dehydration of glycerol over all catalysts was conducted under atmospheric pressure in an automated continuous‐flow fixed‐bed reactor (8 mm inner diameter). A feed of 20 wt % aqueous solution of glycerol was pumped into the reactor by using a metered pump. The reaction temperature was set at 320 °C, and the weight hourly space velocity (WHSV) value was 2.4 h^−1^. The catalyst samples (500 mg, 40–60 mesh sieve fraction) were packed in the central part of reactor between two plugs of quartz sand. Prior to reaction, the catalyst was activated with N_2_ for 2 h at 320 °C. The reaction products were condensed in a cold trap and collected hourly for analysis on a gas chromatograph (Thermo TRACE 1310) equipped with a flame ionization detector (FID) and a TR‐Wax capillary column (30 m×0.25 mm). The conversion of glycerol and the product selectivity were calculated by their peak areas, and compared with known standards.

## Conflict of interest

*The authors declare no conflict of interest*.

## Supporting information

As a service to our authors and readers, this journal provides supporting information supplied by the authors. Such materials are peer reviewed and may be re‐organized for online delivery, but are not copy‐edited or typeset. Technical support issues arising from supporting information (other than missing files) should be addressed to the authors.

Supplementary
