# Supplementary material for: Facile Synthesis of a Novel Hierarchical ZSM‐5 Zeolite: A Stable Acid Catalyst for Dehydrating Glycerol to Acrolein
Source: ChemCatChem. 2017 Oct 16;10(1):211–21. doi: 10.1002/cctc.201700663 (PMC5768019; doi:10.1002/cctc.201700663)
Supplement: Supplementary file 1 — Supplementary [file CCTC-10-211-s001.pdf]

Heterogeneous & Homogeneous & Bio- & Nano-
CHEMCATCHEM
CATALYSIS

Supporting Information

Facile Synthesis of a Novel Hierarchical ZSM-5 Zeolite: A Stable Acid Catalyst for Dehydrating Glycerol to Acrolein

Rolf Beerthuis+,[a] Liang Huang+,[b] N. Raveendran Shiju,[a] Gadi Rothenberg,*[a] Wei Shen,[b] and Hualong Xu*[b]

cctc_201700663_sm_miscellaneous_information.pdf

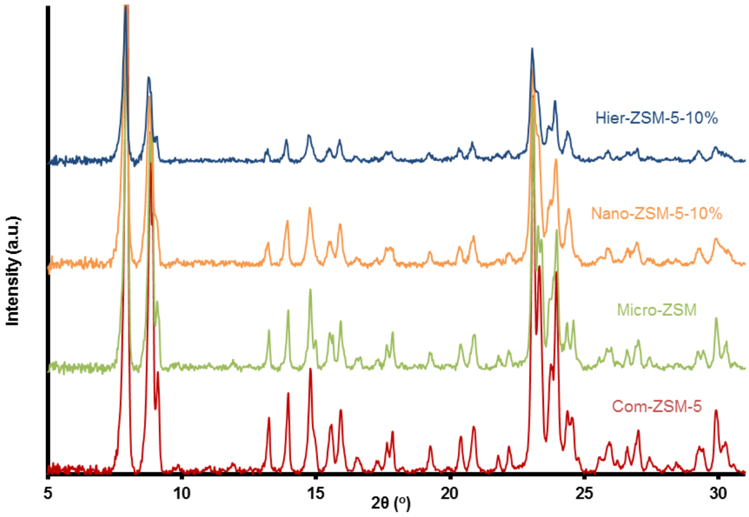

Fig. S1 XRD diffractograms of ZSM-5 samples with varied crystal size.

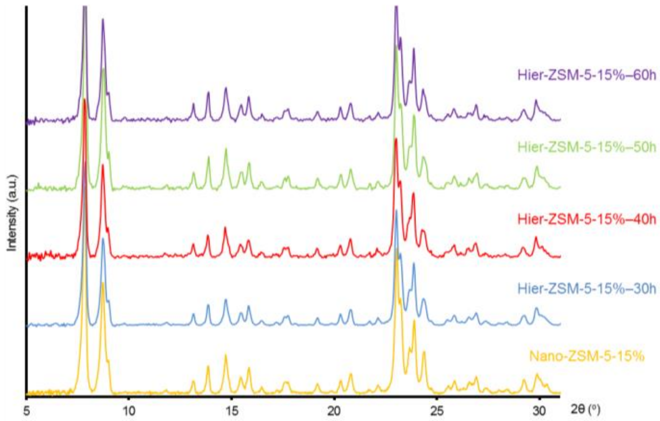

Fig. S2 XRD diffractograms of ZSM-5 samples with optimized crystallization conditions.

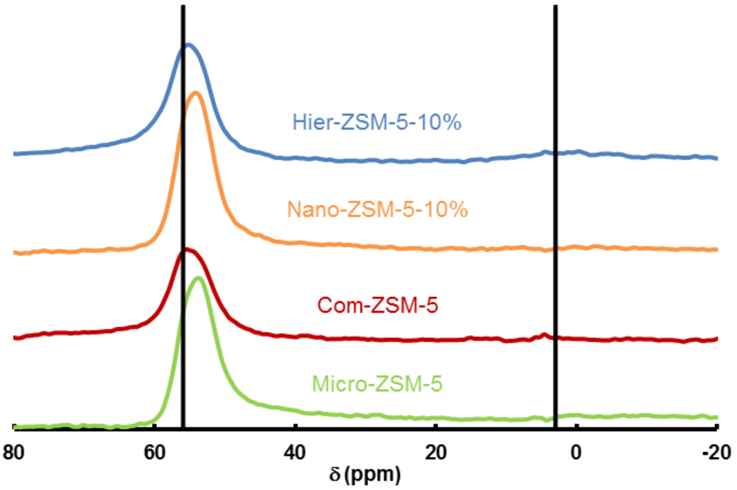

Fig. S3 Solid state ^{27}Al NMR spectra for ZSM-5 samples with varied crystal size.

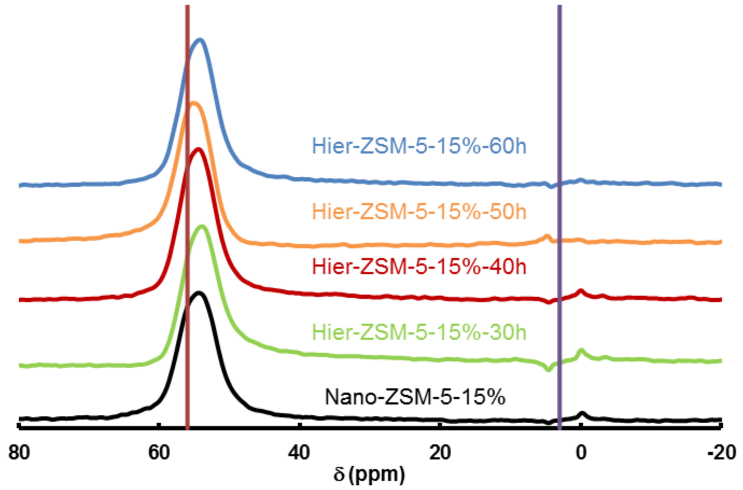

Fig. S4 Solid state ^{27}Al NMR spectra for ZSM-5 samples with optimized crystallization conditions.

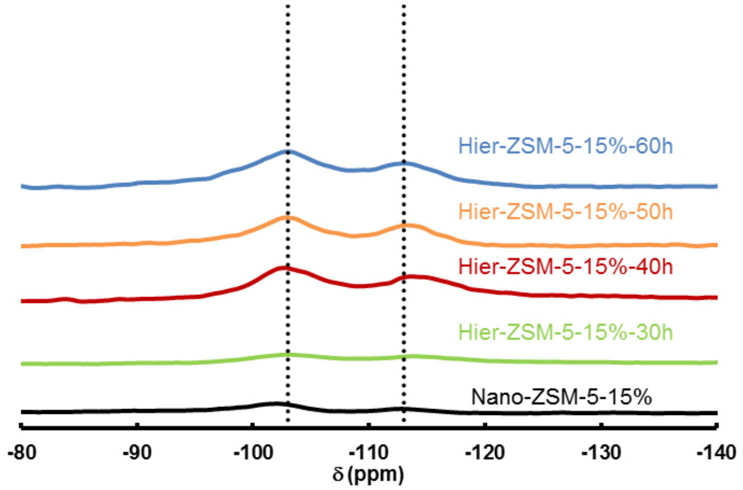

Fig. S5 Solid state ^{29}Si NMR spectra for ZSM-5 samples with optimized crystallization conditions.

Table S1. Physical properties of Hier-ZSM-5-15% samples, after 20 min ultrasonic treatment at 50 Hz.

**Sample**	**Hydrothermal reaction time (h)**	**SA_{BET} (m^2 g^{-1})**	**$SA_{external}$ (m^2 g^{-1})**	**V_{micro} (cm^3 g^{-1})**	**V_{total} (cm^3 g^{-1})**
Hier-ZSM-5-15%	30	347	177	0.08	0.31
Hier-ZSM-5-15%	40	345	169	0.09	0.26
Hier-ZSM-5-15%	50	339	184	0.08	0.30
Hier-ZSM-5-15%	60	330	171	0.08	0.33

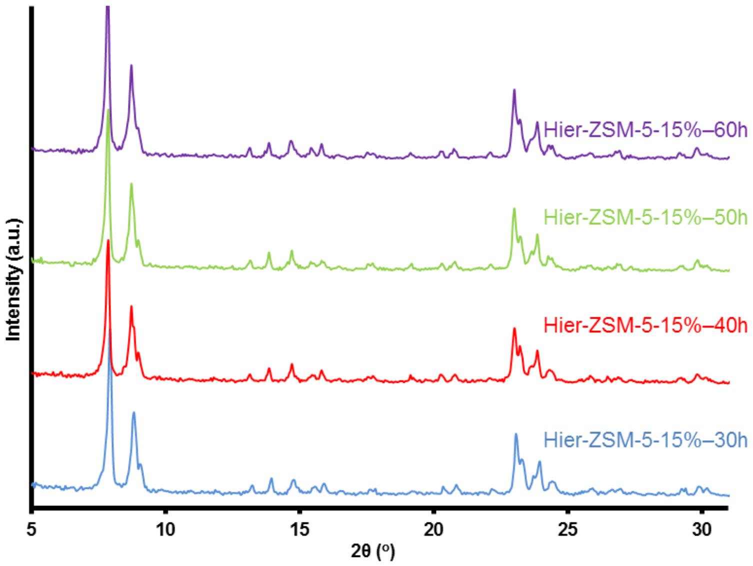

Fig. S6 XRD diffractograms of Hier-ZSM-5-15% samples, after 20 min ultrasonic treatment at 50 Hz.
